# Emerging Roles of Synapse Organizers in the Regulation of Critical Periods

**DOI:** 10.1155/2019/1538137

**Published:** 2019-09-03

**Authors:** Adema Ribic, Thomas Biederer

**Affiliations:** ^1^Department of Neuroscience, Tufts University School of Medicine, Boston, MA 02111, USA; ^2^Department of Neurology, Yale University School of Medicine, New Haven, CT 06511, USA

## Abstract

Experience remodels cortical connectivity during developmental windows called critical periods. Experience-dependent regulation of synaptic strength during these periods establishes circuit functions that are stabilized as critical period plasticity wanes. These processes have been extensively studied in the developing visual cortex, where critical period opening and closure are orchestrated by the assembly, maturation, and strengthening of distinct synapse types. The synaptic specificity of these processes points towards the involvement of distinct molecular pathways. Attractive candidates are pre- and postsynaptic transmembrane proteins that form adhesive complexes across the synaptic cleft. These synapse-organizing proteins control synapse development and maintenance and modulate structural and functional properties of synapses. Recent evidence suggests that they have pivotal roles in the onset and closure of the critical period for vision. In this review, we describe roles of synapse-organizing adhesion molecules in the regulation of visual critical period plasticity and we discuss the potential they offer to restore circuit functions in amblyopia and other neurodevelopmental disorders.

## 1. Introduction

Sensitive periods for the development of brain function have been described in different species and brain areas, but it was the work of Hubel and Wiesel in cat and primate visual cortexes during the 1970s and 1980s that first shed light on the underlying circuit principles [1–4]. This enabled studies of cellular mechanisms, leading to the recognition of synapses in the visual cortex as cellular substrates for critical period plasticity [5–9]. These studies showed that balanced visual input is accompanied by stereotypic developmental remodeling and pruning of synapses in the primary visual cortex, whereas visual deprivation results in synapse loss and shrinkage of axonal and dendritic arbors [5, 10–17]. The application of genetic, chemo-, and optogenetic tools in mice later revealed how vision shapes cortical connectivity during development and how the establishment of cortical connectivity instructs visual function [18–23]. These approaches have also shed light on synaptic mechanisms that control critical periods and actively restrict plasticity in the adult brain [18, 19]. This review is focused on the recently discovered roles of molecules that specify and assemble synaptic connectivity in the onset and closure of plasticity in the visual cortex, a model of cortical plasticity.

## 2. Synaptic Control of Critical Period Timing

**Figure 1:**
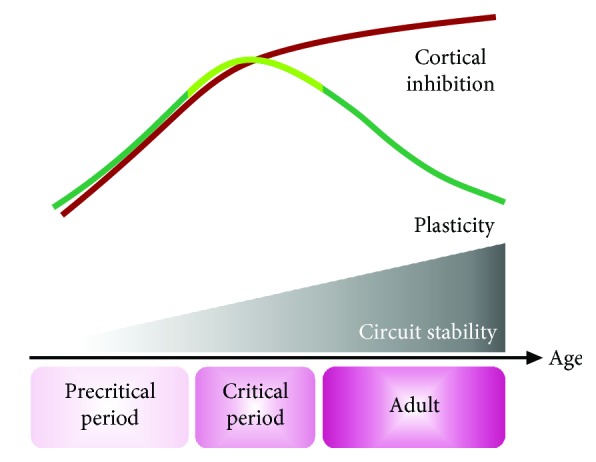
Circuit plasticity, stability, and levels of inhibition as functions of age. Circuit functions are shaped by external experiences during the critical period, when plasticity is high. Levels of cortical inhibitory neurotransmission rise through the critical period and, once optimal function is reached, contribute to the waning of plasticity and stabilization of circuit function in adults.

Circuit functions emerge early in development and are shaped by the environment and patterns of activity during critical periods [24–27]. Heightened plasticity and adaptability of circuits during critical periods enable sensory input, vision included, to guide selective strengthening and refinement of different synapse types [22, 28]. This experience-dependent synaptic remodeling stabilizes the synaptic connectivity patterns that underlie mature circuit function. Notably, in the visual cortex, GABA(gamma-aminobutyric acid)-releasing inhibitory neurons are considered key for critical period timing [29–31]. The onset of synaptic integration of inhibitory neurons into local networks coincides with a rise in inhibitory synapse density and overall levels of inhibitory neurotransmitters in the brain [13, 22, 32–35]. A threshold level of cortical inhibition is necessary for the visual critical period to open, and manipulating GABAergic transmission with pharmacologic or genetic tools can either advance or prevent critical period opening [29–31]. As levels of cortical inhibition further rise in the maturing brain, the critical period closes and the potential for plasticity and remodeling wanes (Figure 1). In parallel, glutamatergic synapses onto both excitatory pyramidal and inhibitory neurons undergo vision-driven remodeling [22, 36]. The heightened circuit plasticity that is characteristic of critical periods is no longer present once mature circuit functions are established, and active stabilization and maintenance of function take over in the adult brain [18, 24, 26, 27] (Figure 1).

High levels of inhibition in adults are thought to contribute to the stabilization of mature brain function by limiting circuit plasticity (Figure 1) [24]. Indeed, acute reduction in levels of inhibitory neurotransmitters in the mature visual cortex can reinstate visual plasticity [37, 38]. On a cellular level, manipulation of activity of soma-targeting, fast-spiking Parvalbumin (PV) and dendrite-targeting, regular-spiking Somatostatin (SST) circuitry results in robust changes in visual plasticity [18, 39–47]. These interneuron classes exert powerful control over critical period onset: transplantation of embryonic PV and SST interneurons derived from medial ganglionic eminence into the adult visual cortex can trigger another visual critical period, with remarkably preserved timing of onset and closure [40, 48]. These precise developmental sequences indicate tight genetic control of interneuron maturation, which is well described for PV interneurons [49–52]. PV interneuron maturation is directed, at least in part, by the complex interplay of Orthodenticle Homeobox 2 (Otx2), a non-cell-autonomous transcription factor secreted from the retina and choroid plexus, and the extracellular matrix (ECM) deposited around interneurons [50, 51, 53–57]. The capture of Otx2 by the ECM that surrounds PV interneurons is essential for the onset of their maturation [57, 58], and misregulated Otx2 expression and localization lead to deficits in critical period plasticity [50, 51, 53, 57–60]. The stereotypic circuit integration of transplanted PV interneurons supports the additional involvement of cell-autonomous factors that control the development of synaptic connectivity of these cells [48]. Activity-driven assembly of local excitatory inputs onto PV interneurons prior to critical period opening in mice is pivotal for its onset [19]. The parallel increase in interneuron expression of synapse-organizing adhesion proteins such as Neuroligins and SynCAMs (see below) further supports that synaptogenesis is an important factor in PV cell maturation [61]. A recent study demonstrated that PV interneuron-expressed Synaptic Cell Adhesion Molecule 1 (SynCAM 1) is required for critical period closure, which involves the SynCAM 1-dependent formation of long-range excitatory inputs from the thalamus [18]. In the following sections, we describe known molecular regulators of synaptic connectivity in the visual cortex.

## 3. Roles of Synapse-Organizing Proteins in Visual Cortex Synaptogenesis and Plasticity

Cell adhesion proteins that instruct synapse assembly and their maintenance are expressed in diverse neuron types and in glial cells [62–66]. These proteins were initially identified as potent drivers of presynaptic differentiation in an *in vitro* heterologous system, and they form complexes in *trans* (for adhesion) and in *cis* (for lateral assembly) [66–70]. After instructing the assembly of pre- and postsynaptic specializations into functional synapses, these proteins can maintain synapses in the maturing brain [71–73]. Recent research suggests that distinct pairs of synaptic organizers impact different synapse types in the cortex [74, 75] as summarized below.

### 3.1. Neuroligins and Hevin

**Figure 2:**
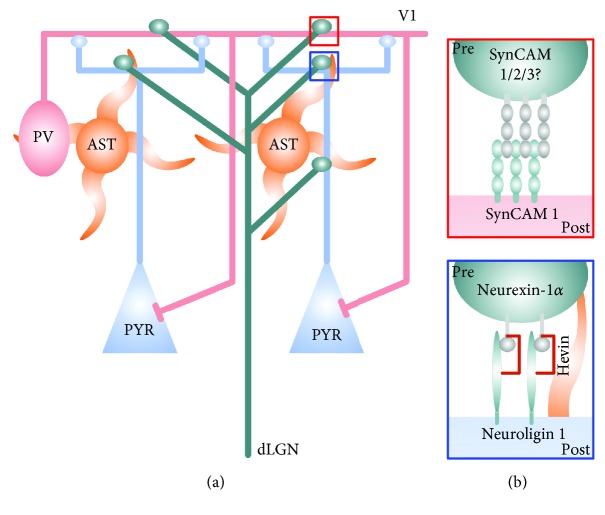
Synaptic connectivity of the visual thalamocortical circuit. (a) Excitatory inputs carrying visual information from the dorsal lateral geniculate nucleus (dLGN, green) in the thalamus innervate pyramidal (PYR, blue box) neurons and Parvalbumin (PV, red box) interneurons in thalamorecipient layers of the visual cortex (red box). PV interneurons receive inputs from neighbouring PYR neurons across cortical layers. Astrocytes (AST) express molecules that can act as synaptic bridges between thalamocortical axons and their postsynaptic targets (Hevin, blue box). (b) Red box: the synaptic immunoglobulin SynCAM 1 organizes thalamic inputs onto PV interneurons. Presynaptic interacting partners of SynCAM 1 at thalamocortical synapses are currently unknown, but other SynCAMs (2 and 3) are candidates. Blue box: Neuroligin 1 on PYR cells interacts with Neurexin-1*α* via the astrocytic Hevin (brown) to organize thalamic inputs onto PYR cells. Astrocytic process is depicted in orange. Presynapse (Pre) and postsynapse (Post) are indicated.

Neuroligins are prototypical postsynaptic synapse organizers and type 1 transmembrane proteins that interact with presynaptic Neurexins [67, 76, 77]. Neuroligins 1-4 are redundant for synapse assembly *in vivo* but are key for synapse maturation and function [65, 77]. Their interactions with *α*- and *β*-Neurexins affect both inhibitory and excitatory presynaptic functions, as well as recruitment of synapse scaffolding components and neurotransmitter receptors to the postsynapse [78–83]. Different combinations of Neuroligin/Neurexin complexes can potentially specify different synapse types, and the repertoire of these interactions is expanded by splicing isoforms [84] and accessory extracellular linker proteins, such as glia-expressed Hevin [85] (Figure 2). While cell-surface expression levels of Neuroligins can be regulated by visual activity [86], it is the removal of Hevin in the visual cortex that impairs Neuroligin 1/Neurexin interaction and reduces the density of thalamic inputs (Figure 2) [85, 87]. Mice that lack Hevin show impaired ocular dominance and critical period opening, suggesting that the assembly of thalamocortical synapses by Neuroligin 1/Neurexin/Hevin interactions controls the opening of the visual critical period [85]. Hevin knockout mice display a compensatory increase in local, intracortical excitatory synapses that is insufficient to open the critical period, indicating that specific synapse types are key for different circuit functions [85].

### 3.2. SynCAMs

Similar to Neuroligins, SynCAM cell adhesion complexes are prominently expressed in the visual cortex and recent research highlighted their role in timing the onset and offset of cortical critical periods [18, 88, 89]. SynCAMs are potent inducers of synapse differentiation *in vitro* [68, 90] that contribute to excitatory synapse formation and maintenance *in vivo* across different brain regions [18, 72, 91, 92]. SynCAMs 1-4 are immunoglobulin domain type-1 transmembrane proteins, whose homo- and heterophilic interactions across the synaptic cleft organize excitatory synapses [90, 93]. The most studied family member is SynCAM 1 that interacts with itself and SynCAMs 2 and 3 in *cis* and *trans* [90, 93–95]. SynCAM 1 controls both pre- and postsynaptic properties through its interactions across the synaptic cleft and affects cytoskeletal remodeling and receptor recruitment at the synapse through its intracellular partners [72, 88, 96, 97]. In the cortex, SynCAM 1 recruits large and potent long-range thalamocortical excitatory inputs onto PV interneurons (Figure 2) [18, 91]. Further, PV-expressed SynCAM 1 is regulated by visual activity [18]. In agreement with its role in PV maturation, SynCAM 1 is a regulatory target of Otx2 [52] and is essential for maturation of PV interneurons in the visual cortex. Similar to Hevin knockout mice, mice that lack SynCAM 1 have fewer thalamocortical synapses (Figure 2) [18]. This results in poorly developed binocular vision and an extended visual critical period [18]. SynCAM 1 is actively required to control plasticity and even a brief cell-specific removal of SynCAM 1 from PV interneurons results in increased levels of visual plasticity in the adult brain, pointing to a key role for thalamic inputs onto PV interneurons in the regulation of plasticity in mature circuits [18]. This cell-autonomous, postsynaptic requirement for SynCAM 1 in PV interneurons suggests that postsynaptic SynCAM 1 engages currently unknown transsynaptic partners in thalamic axons to assemble thalamocortical synapses (Figure 2) [18, 90].

### 3.3. Distinct Roles of Neuroligin/Hevin and SynCAM 1

As reviewed above, both Neuroligin/Neurexin interaction (through Hevin) and SynCAM 1 play a role in the formation of thalamocortical synapses but with opposing effects on visual plasticity [18, 85]. Lack of Hevin prevents the critical period from opening, whereas lack of SynCAM 1 prevents it from closure [18, 85]. However, Hevin appears to affect most, if not all, excitatory thalamocortical synapses formed across neuron types, while SynCAM 1 shows a PV-specific action on thalamocortical inputs [18, 85, 87]. It is possible that gross development of thalamocortical synapses mediated by Neuroligin 1/Neurexin-1*α*/Hevin interaction is a prerequisite for the critical period to open, and PV-specific recruitment and maintenance of thalamic inputs by SynCAM 1 is necessary for subsequent critical period closure. Future studies can address whether any cross-talk between the two pathways exists in PV interneurons, as well as whether these molecules control plasticity through thalamocortical synapses in other sensory or association areas [98, 99].

### 3.4. Extracellular Matrix, LRRTMs, and NCAM

So far, only SynCAMs and Neuroligins (through Hevin) have demonstrated roles in visual plasticity, but recent research demonstrated that members of the leucine-rich repeat transmembrane (LRRTM) family of molecules can interact at synapses with the extracellular matrix (ECM), a powerful regulator of visual plasticity [34, 100]. LRRTMs 1-4 are another group of type 1 transmembrane proteins that bind Neurexins, potently induce excitatory presynaptic differentiation and regulate receptor composition at the synapse [70, 101, 102]. LRRTM-deficient mice show defects in both pre- and postsynaptic functions, and their repertoire of interactions with Neurexins can impact diverse synapse types [70, 74, 103, 104]. LRRTMs bind Neurexins across the synaptic cleft similar to Neuroligins, but they can also instruct differential synapse formation through interactions with components of the ECM [100–102, 105]. As the ECM in the form of perineuronal nets exerts powerful control over the maturation of PV interneurons and critical period timing [34, 58, 106–111], the role of LRRTMs in visual plasticity warrants future investigation. An ECM-related protein modification, the polysialylation of neural cell adhesion molecule (NCAM), guides the development of inhibitory connections in the visual cortex [112]. NCAM is an immunoglobulin superfamily protein that regulates early synapse development and is mostly found in a glycan-bound state [113]. Visual activity-dependent polysialylation of NCAM affects its homophilic interactions across the synapse, and removal of PSA from NCAM can shift the critical period to an earlier time point through modulation of PV connectivity [112]. SynCAM 1 can also be found in the polysialylated state, pointing to yet another way to diversify the function and interactions of synapse organizers [114, 115].

## 4. Therapeutic Potential of Synapse-Organizing Molecules in Amblyopia and Neurodevelopmental Disorders

The diminished plasticity of mature circuits is thought to preclude recovery from early visual insults such as amblyopia. Patching or visual stimulation can provide therapeutic interventions before the critical period closes, but the reduced capacity of visual synapses for activity-driven remodeling likely interferes with the success of interventions later in life [116–118]. The reduced potential of the adult brain to rewire itself may also impede treatments for other neurodevelopmental disorders, such as autism-spectrum disorders (ASD) and schizophrenia [55, 119–122]. Studies of amblyopia and visual plasticity have identified promising interventions for recovering the potential for plasticity in the entire brain, such as neuromodulation of inhibitory connections [46, 123], systemic regulation of inhibitory neurotransmission [124], and sensory manipulations that may target the activity of thalamocortical synapses [125–127]. On a more specific level, recent research has demonstrated that the cell-specific manipulation of thalamocortical synapses reinstates plastic features to the adult visual cortex [18]. As distinct circuits regulate plasticity of binocularity and improvements in visual acuity in amblyopia models [128, 129], targeting synapses that organize different circuits may hence represent a way to precisely manipulate different brain functions.

How do we target specific synapse types? Transient genetic silencing tools in combination with cell-specific adenoviral vectors could allow manipulating synapse organizers in a cell type-and-region-specific manner [130–132]. Further, peptide fragments of extracellular domains of synapse organizers can impair their interactions *in vitro* and may have a similar effect *in vivo* [86, 93]. Indeed, a recent study using a combination of these approaches to manipulate signaling by a secreted molecule, semaphorin 3A, demonstrated its feasibility in rat models of amblyopia [133]. Such approaches may increase plasticity to a level sufficient for visual therapy to have effects in adult amblyopic patients [116–118, 133–136]. These tools could provide a localized therapy that can be restricted to the visual cortex alone, thus precluding systemic side-effects. A transient elevation of cortical plasticity may even improve therapeutic outcomes for other neurodevelopmental disorders [137–140]. Approaches that result in the elevated potential for plasticity in the mature brain could additionally enhance recovery after brain injury, including traumatic brain injury (TBI) and stroke [120, 141–147]. In combination with targeting mechanisms that control neuronal specification [148–152], tools that target specific synapse types hence offer highly specific therapeutic interventions for developmental brain disorders. Future studies on mechanisms of synapse specification within distinct circuits are likely to provide an avenue for progress in this area.
